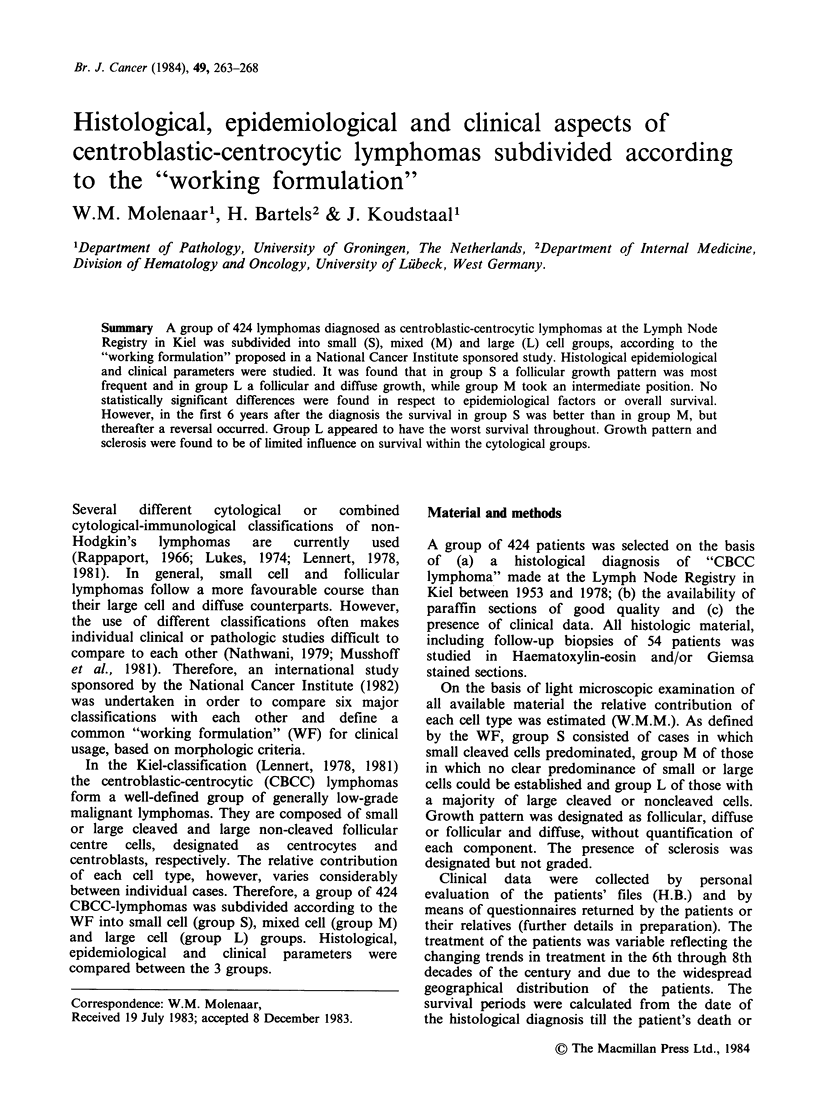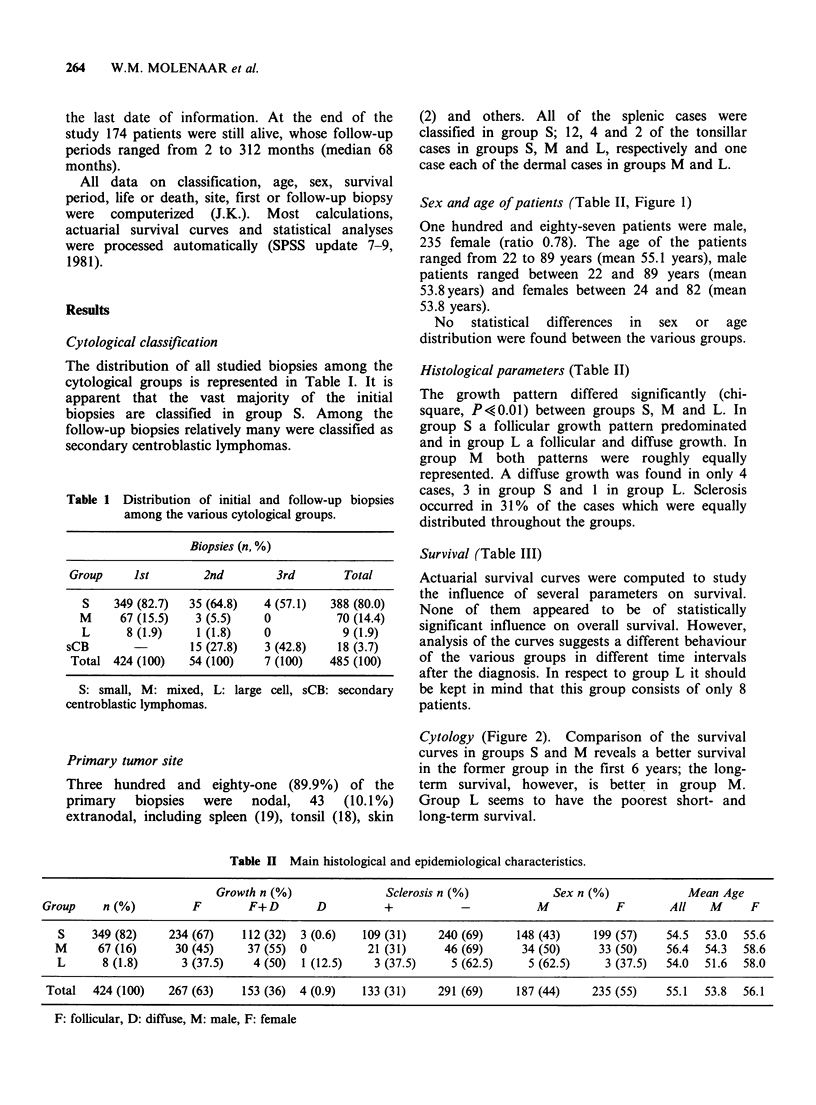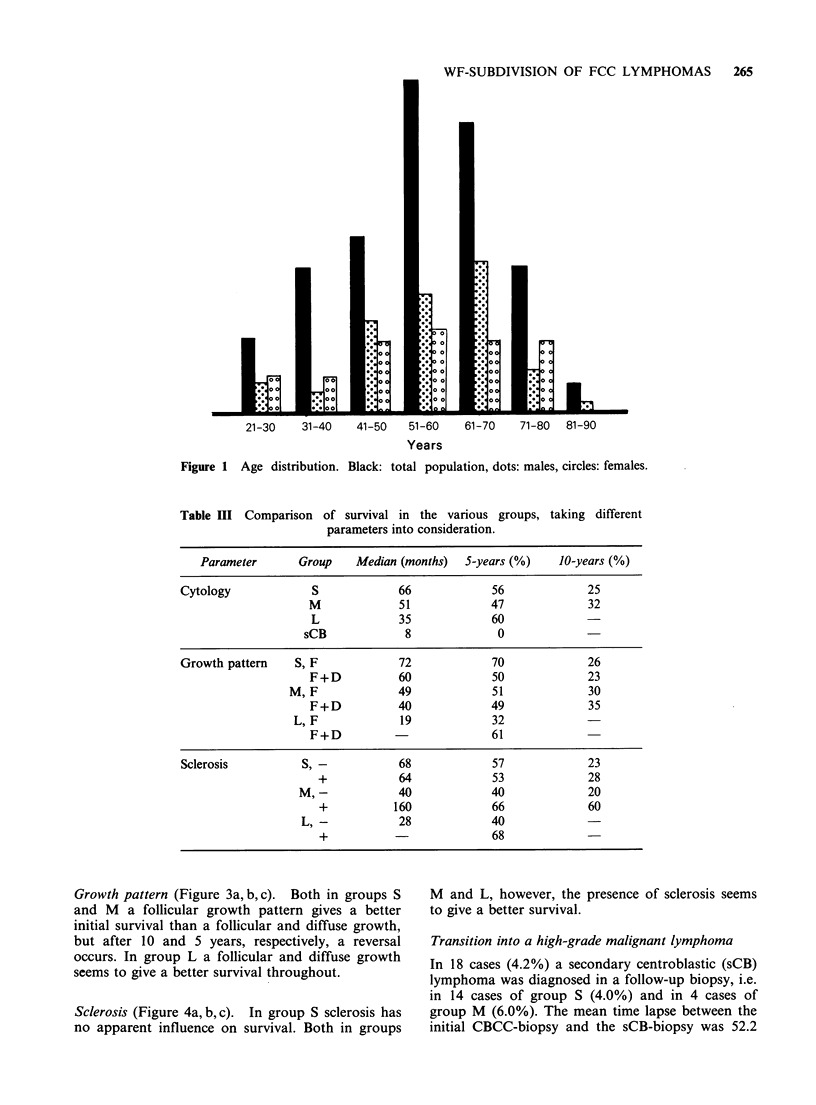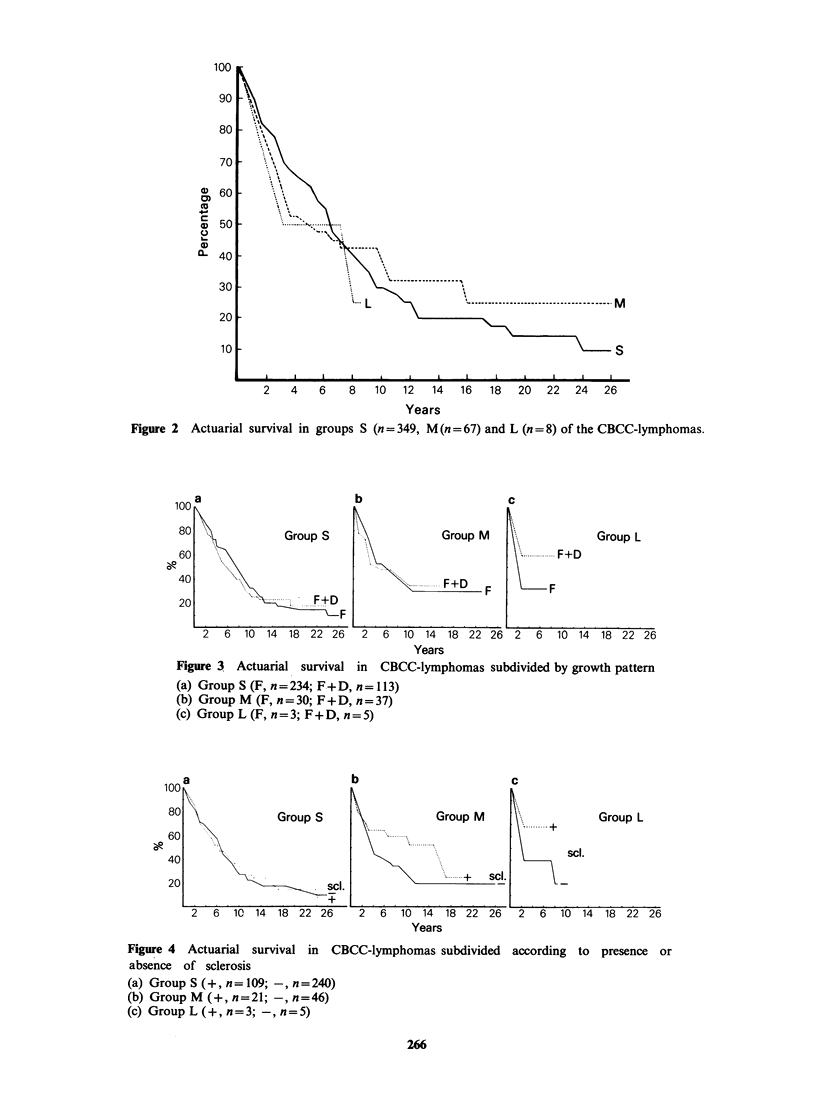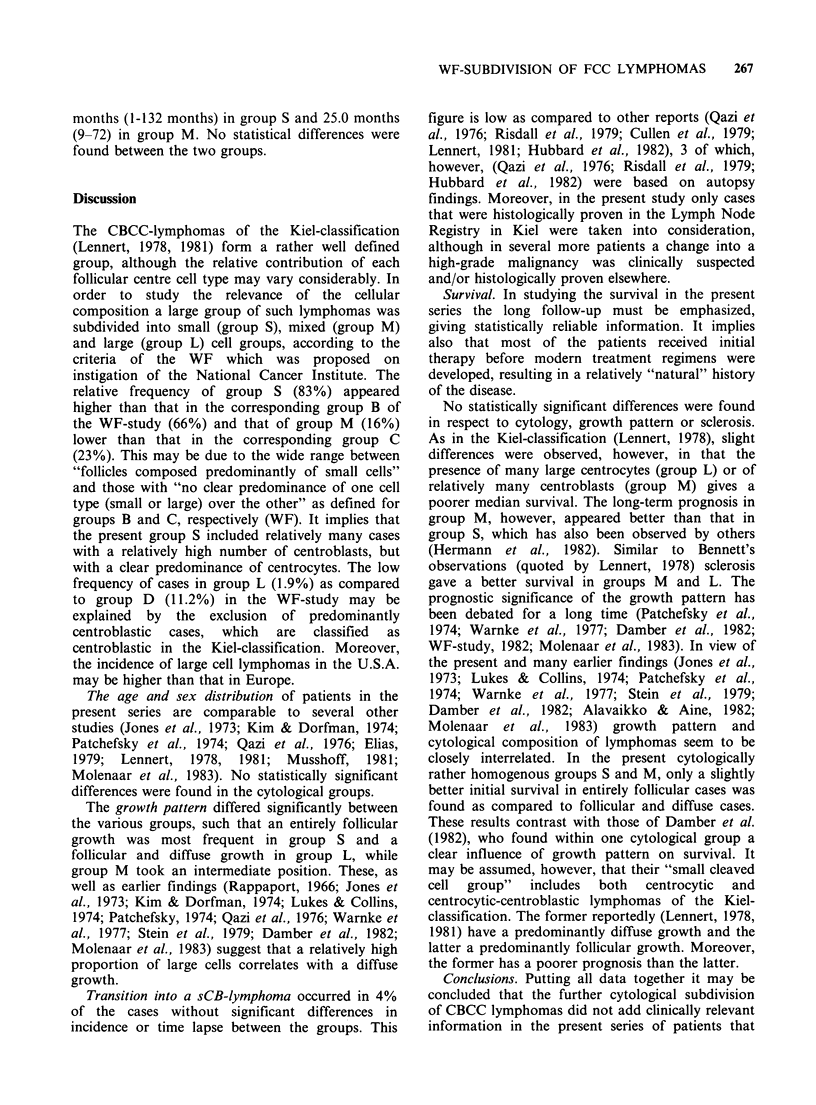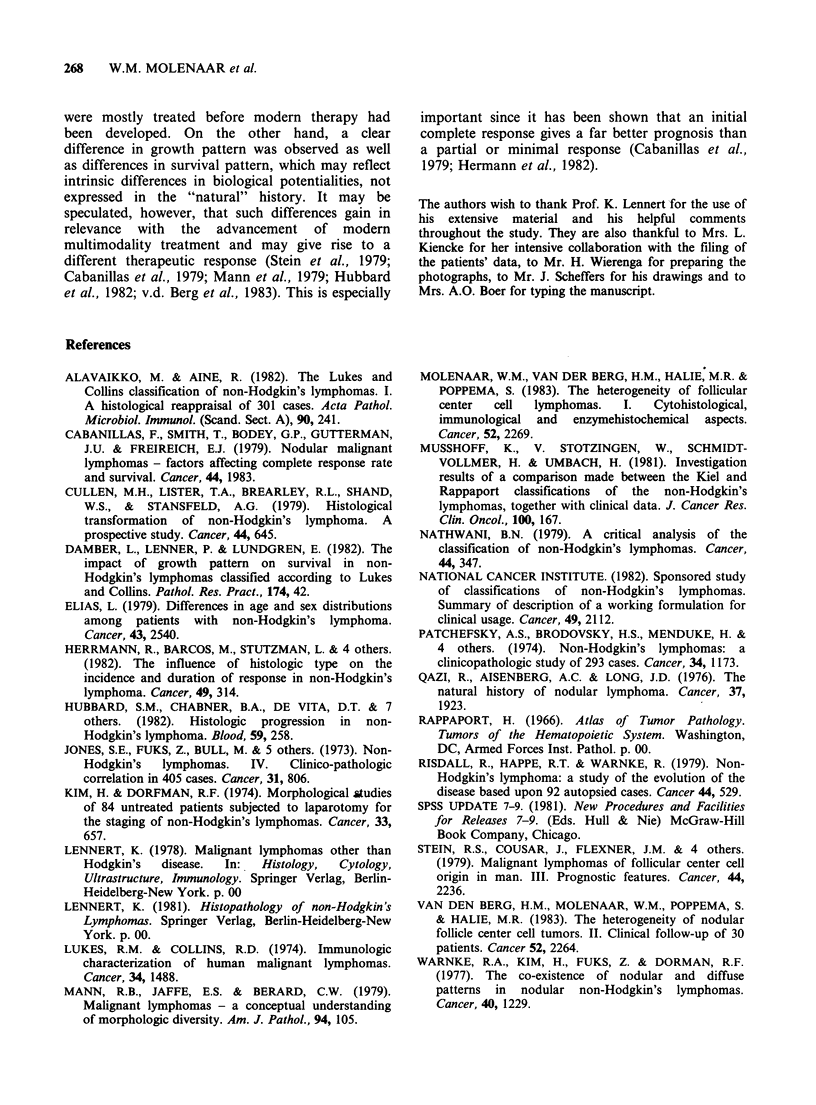# Histological, epidemiological and clinical aspects of centroblastic-centrocytic lymphomas subdivided according to the "working formulation".

**DOI:** 10.1038/bjc.1984.43

**Published:** 1984-03

**Authors:** W. M. Molenaar, H. Bartels, J. Koudstaal

## Abstract

A group of 424 lymphomas diagnosed as centroblastic-centrocytic lymphomas at the Lymph Node Registry in Kiel was subdivided into small (S), mixed (M) and large (L) cell groups, according to the "working formulation" proposed in a National Cancer Institute sponsored study. Histological epidemiological and clinical parameters were studied. It was found that in group S a follicular growth pattern was most frequent and in group L a follicular and diffuse growth, while group M took an intermediate position. No statistically significant differences were found in respect to epidemiological factors or overall survival. However, in the first 6 years after the diagnosis the survival in group S was better than in group M, but thereafter a reversal occurred. Group L appeared to have the worst survival throughout. Growth pattern and sclerosis were found to be of limited influence on survival within the cytological groups.


*Br. J. Cancer* (1984), **49**, 263–268

### Histological, epidemiological and clinical aspects of centroblastic-centrocytic lymphomas subdivided according to the "working formulation"

W.M. Molenaar[1], H. Bartels[2] & J. Koudstaal[1]

[1]*Department of Pathology, University of Groningen, The Netherlands,* [2]*Department of Internal Medicine, Division of Hematology and Oncology, University of Lübeck, West Germany.*

**Summary** A group of 424 lymphomas diagnosed as centroblastic-centrocytic lymphomas at the Lymph Node Registry in Kiel was subdivided into small (S), mixed (M) and large (L) cell groups, according to the "working formulation" proposed in a National Cancer Institute sponsored study. Histological epidemiological and clinical parameters were studied. It was found that in group S a follicular growth pattern was most frequent and in group L a follicular and diffuse growth, while group M took an intermediate position. No statistically significant differences were found in respect to epidemiological factors or overall survival. However, in the first 6 years after the diagnosis the survival in group S was better than in group M, but thereafter a reversal occurred. Group L appeared to have the worst survival throughout. Growth pattern and sclerosis were found to be of limited influence on survival within the cytological groups.

Several different cytological or combined cytological-immunological classifications of non-Hodgkin's lymphomas are currently used (Rappaport, 1966; Lukes, 1974; Lennert, 1978, 1981). In general, small cell and follicular lymphomas follow a more favourable course than their large cell and diffuse counterparts. However, the use of different classifications often makes individual clinical or pathologic studies difficult to compare to each other (Nathwani, 1979; Musshoff *et al.,* 1981). Therefore, an international study sponsored by the National Cancer Institute (1982) was undertaken in order to compare six major classifications with each other and define a common "working formulation" (WF) for clinical usage, based on morphologic criteria.

In the Kiel-classification (Lennert, 1978, 1981) the centroblastic-centrocytic (CBCC) lymphomas form a well-defined group of generally low-grade malignant lymphomas. They are composed of small or large cleaved and large non-cleaved follicular centre cells, designated as centrocytes and centroblasts, respectively. The relative contribution of each cell type, however, varies considerably between individual cases. Therefore, a group of 424 CBCC-lymphomas was subdivided according to the WF into small cell (group S), mixed cell (group M) and large cell (group L) groups. Histological, epidemiological and clinical parameters were compared between the 3 groups.

Correspondence: W.M. Molenaar,
Received 19 July 1983; accepted 8 December 1983.

### Material and methods

A group of 424 patients was selected on the basis of (a) a histological diagnosis of "CBCC lymphoma" made at the Lymph Node Registry in Kiel between 1953 and 1978; (b) the availability of paraffin sections of good quality and (c) the presence of clinical data. All histologic material, including follow-up biopsies of 54 patients was studied in Haematoxylin-eosin and/or Giemsa stained sections.

On the basis of light microscopic examination of all available material the relative contribution of each cell type was estimated (W.M.M.). As defined by the WF, group S consisted of cases in which small cleaved cells predominated, group M of those in which no clear predominance of small or large cells could be established and group L of those with a majority of large cleaved or noncleaved cells. Growth pattern was designated as follicular, diffuse or follicular and diffuse, without quantification of each component. The presence of sclerosis was designated but not graded.

Clinical data were collected by personal evaluation of the patients' files (H.B.) and by means of questionnaires returned by the patients or their relatives (further details in preparation). The treatment of the patients was variable reflecting the changing trends in treatment in the 6th through 8th decades of the century and due to the widespread geographical distribution of the patients. The survival periods were calculated from the date of the histological diagnosis till the patient's death or

© The Macmillan Press Ltd., 1984

the last date of information. At the end of the study 174 patients were still alive, whose follow-up periods ranged from 2 to 312 months (median 68 months).

All data on classification, age, sex, survival period, life or death, site, first or follow-up biopsy were computerized (J.K.). Most calculations, actuarial survival curves and statistical analyses were processed automatically (SPSS update 7–9, 1981).

### Results

### *Cytological classification*

The distribution of all studied biopsies among the cytological groups is represented in Table I. It is apparent that the vast majority of the initial biopsies are classified in group S. Among the follow-up biopsies relatively many were classified as secondary centroblastic lymphomas.

**Table 1** Distribution of initial and follow-up biopsies among the various cytological groups.

| | *Biopsies (n, %)* | | | |
|---|---|---|---|---|
| *Group* | *1st* | *2nd* | *3rd* | *Total* |
| S | 349 (82.7) | 35 (64.8) | 4 (57.1) | 388 (80.0) |
| M | 67 (15.5) | 3 (5.5) | 0 | 70 (14.4) |
| L | 8 (1.9) | 1 (1.8) | 0 | 9 (1.9) |
| sCB | — | 15 (27.8) | 3 (42.8) | 18 (3.7) |
| Total | 424 (100) | 54 (100) | 7 (100) | 485 (100) |

S: small, M: mixed, L: large cell, sCB: secondary centroblastic lymphomas.

### *Primary tumor site*

Three hundred and eighty-one (89.9%) of the primary biopsies were nodal, 43 (10.1%) extranodal, including spleen (19), tonsil (18), skin (2) and others. All of the splenic cases were classified in group S; 12, 4 and 2 of the tonsillar cases in groups S, M and L, respectively and one case each of the dermal cases in groups M and L.

### *Sex and age of patients (*Table II, Figure 1)

One hundred and eighty-seven patients were male, 235 female (ratio 0.78). The age of the patients ranged from 22 to 89 years (mean 55.1 years), male patients ranged between 22 and 89 years (mean 53.8 years) and females between 24 and 82 (mean 53.8 years).

No statistical differences in sex or age distribution were found between the various groups.

### *Histological parameters* (Table II)

The growth pattern differed significantly (chi-square, $P \ll 0.01$) between groups S, M and L. In group S a follicular growth pattern predominated and in group L a follicular and diffuse growth. In group M both patterns were roughly equally represented. A diffuse growth was found in only 4 cases, 3 in group S and 1 in group L. Sclerosis occurred in 31% of the cases which were equally distributed throughout the groups.

### *Survival (*Table III)

Actuarial survival curves were computed to study the influence of several parameters on survival. None of them appeared to be of statistically significant influence on overall survival. However, analysis of the curves suggests a different behaviour of the various groups in different time intervals after the diagnosis. In respect to group L it should be kept in mind that this group consists of only 8 patients.

*Cytology* (Figure 2). Comparison of the survival curves in groups S and M reveals a better survival in the former group in the first 6 years; the long-term survival, however, is better in group M. Group L seems to have the poorest short- and long-term survival.

**Table II** Main histological and epidemiological characteristics.

| | | *Growth n (%)* | | | *Sclerosis n (%)* | | *Sex n (%)* | | *Mean Age* | | |
|---|---|---|---|---|---|---|---|---|---|---|---|
| *Group* | *n (%)* | *F* | *F+D* | *D* | *+* | *–* | *M* | *F* | *All* | *M* | *F* |
| S | 349 (82) | 234 (67) | 112 (32) | 3 (0.6) | 109 (31) | 240 (69) | 148 (43) | 199 (57) | 54.5 | 53.0 | 55.6 |
| M | 67 (16) | 30 (45) | 37 (55) | 0 | 21 (31) | 46 (69) | 34 (50) | 33 (50) | 56.4 | 54.3 | 58.6 |
| L | 8 (1.8) | 3 (37.5) | 4 (50) | 1 (12.5) | 3 (37.5) | 5 (62.5) | 5 (62.5) | 3 (37.5) | 54.0 | 51.6 | 58.0 |
| Total | 424 (100) | 267 (63) | 153 (36) | 4 (0.9) | 133 (31) | 291 (69) | 187 (44) | 235 (55) | 55.1 | 53.8 | 56.1 |

F: follicular, D: diffuse, M: male, F: female

**Figure 1** Age distribution. Black: total population, dots: males, circles: females.

**Table III** Comparison of survival in the various groups, taking different parameters into consideration.

| *Parameter* | *Group* | *Median (months)* | *5-years (%)* | *10-years (%)* |
|---|---|---|---|---|
| Cytology | S | 66 | 56 | 25 |
| | M | 51 | 47 | 32 |
| | L | 35 | 60 | — |
| | sCB | 8 | 0 | — |
| Growth pattern | S, F | 72 | 70 | 26 |
| | F+D | 60 | 50 | 23 |
| | M, F | 49 | 51 | 30 |
| | F+D | 40 | 49 | 35 |
| | L, F | 19 | 32 | — |
| | F+D | — | 61 | — |
| Sclerosis | S, − | 68 | 57 | 23 |
| | + | 64 | 53 | 28 |
| | M, − | 40 | 40 | 20 |
| | + | 160 | 66 | 60 |
| | L, − | 28 | 40 | — |
| | + | — | 68 | — |

*Growth pattern* (Figure 3a, b, c). Both in groups S and M a follicular growth pattern gives a better initial survival than a follicular and diffuse growth, but after 10 and 5 years, respectively, a reversal occurs. In group L a follicular and diffuse growth seems to give a better survival throughout.

*Sclerosis* (Figure 4a, b, c). In group S sclerosis has no apparent influence on survival. Both in groups M and L, however, the presence of sclerosis seems to give a better survival.

*Transition into a high-grade malignant lymphoma*

In 18 cases (4.2%) a secondary centroblastic (sCB) lymphoma was diagnosed in a follow-up biopsy, i.e. in 14 cases of group S (4.0%) and in 4 cases of group M (6.0%). The mean time lapse between the initial CBCC-biopsy and the sCB-biopsy was 52.2

**Figure 2** Actuarial survival in groups S ($n=349$, M($n=67$) and L ($n=8$) of the CBCC-lymphomas.

**Figure 3** Actuarial survival in CBCC-lymphomas subdivided by growth pattern

(a) Group S (F, $n=234$; F+D, $n=113$)
(b) Group M (F, $n=30$; F+D, $n=37$)
(c) Group L (F, $n=3$; F+D, $n=5$)

**Figure 4** Actuarial survival in CBCC-lymphomas subdivided according to presence or absence of sclerosis

(a) Group S (+, $n=109$; −, $n=240$)
(b) Group M (+, $n=21$; −, $n=46$)
(c) Group L (+, $n=3$; −, $n=5$)

months (1-132 months) in group S and 25.0 months (9–72) in group M. No statistical differences were found between the two groups.

### Discussion

The CBCC-lymphomas of the Kiel-classification (Lennert, 1978, 1981) form a rather well defined group, although the relative contribution of each follicular centre cell type may vary considerably. In order to study the relevance of the cellular composition a large group of such lymphomas was subdivided into small (group S), mixed (group M) and large (group L) cell groups, according to the criteria of the WF which was proposed on instigation of the National Cancer Institute. The relative frequency of group S (83%) appeared higher than that in the corresponding group B of the WF-study (66%) and that of group M (16%) lower than that in the corresponding group C (23%). This may be due to the wide range between "follicles composed predominantly of small cells" and those with "no clear predominance of one cell type (small or large) over the other" as defined for groups B and C, respectively (WF). It implies that the present group S included relatively many cases with a relatively high number of centroblasts, but with a clear predominance of centrocytes. The low frequency of cases in group L (1.9%) as compared to group D (11.2%) in the WF-study may be explained by the exclusion of predominantly centroblastic cases, which are classified as centroblastic in the Kiel-classification. Moreover, the incidence of large cell lymphomas in the U.S.A. may be higher than that in Europe.

*The age and sex distribution* of patients in the present series are comparable to several other studies (Jones *et al.*, 1973; Kim & Dorfman, 1974; Patchefsky *et al.*, 1974; Qazi *et al.*, 1976; Elias, 1979; Lennert, 1978, 1981; Musshoff, 1981; Molenaar *et al.*, 1983). No statistically significant differences were found in the cytological groups.

The *growth pattern* differed significantly between the various groups, such that an entirely follicular growth was most frequent in group S and a follicular and diffuse growth in group L, while group M took an intermediate position. These, as well as earlier findings (Rappaport, 1966; Jones *et al.*, 1973; Kim & Dorfman, 1974; Lukes & Collins, 1974; Patchefsky, 1974; Qazi *et al.*, 1976; Warnke *et al.*, 1977; Stein *et al.*, 1979; Damber *et al.*, 1982; Molenaar *et al.*, 1983) suggest that a relatively high proportion of large cells correlates with a diffuse growth.

*Transition into a sCB-lymphoma* occurred in 4% of the cases without significant differences in incidence or time lapse between the groups. This figure is low as compared to other reports (Qazi *et al.*, 1976; Risdall *et al.*, 1979; Cullen *et al.*, 1979; Lennert, 1981; Hubbard *et al.*, 1982), 3 of which, however, (Qazi *et al.*, 1976; Risdall *et al.*, 1979; Hubbard *et al.*, 1982) were based on autopsy findings. Moreover, in the present study only cases that were histologically proven in the Lymph Node Registry in Kiel were taken into consideration, although in several more patients a change into a high-grade malignancy was clinically suspected and/or histologically proven elsewhere.

*Survival.* In studying the survival in the present series the long follow-up must be emphasized, giving statistically reliable information. It implies also that most of the patients received initial therapy before modern treatment regimens were developed, resulting in a relatively "natural" history of the disease.

No statistically significant differences were found in respect to cytology, growth pattern or sclerosis. As in the Kiel-classification (Lennert, 1978), slight differences were observed, however, in that the presence of many large centrocytes (group L) or of relatively many centroblasts (group M) gives a poorer median survival. The long-term prognosis in group M, however, appeared better than that in group S, which has also been observed by others (Hermann *et al.*, 1982). Similar to Bennett's observations (quoted by Lennert, 1978) sclerosis gave a better survival in groups M and L. The prognostic significance of the growth pattern has been debated for a long time (Patchefsky *et al.*, 1974; Warnke *et al.*, 1977; Damber *et al.*, 1982; WF-study, 1982; Molenaar *et al.*, 1983). In view of the present and many earlier findings (Jones *et al.*, 1973; Lukes & Collins, 1974; Patchefsky *et al.*, 1974; Warnke *et al.*, 1977; Stein *et al.*, 1979; Damber *et al.*, 1982; Alavaikko & Aine, 1982; Molenaar *et al.*, 1983) growth pattern and cytological composition of lymphomas seem to be closely interrelated. In the present cytologically rather homogenous groups S and M, only a slightly better initial survival in entirely follicular cases was found as compared to follicular and diffuse cases. These results contrast with those of Damber *et al.* (1982), who found within one cytological group a clear influence of growth pattern on survival. It may be assumed, however, that their "small cleaved cell group" includes both centrocytic and centrocytic-centroblastic lymphomas of the Kiel-classification. The former reportedly (Lennert, 1978, 1981) have a predominantly diffuse growth and the latter a predominantly follicular growth. Moreover, the former has a poorer prognosis than the latter.

*Conclusions.* Putting all data together it may be concluded that the further cytological subdivision of CBCC lymphomas did not add clinically relevant information in the present series of patients that were mostly treated before modern therapy had been developed. On the other hand, a clear difference in growth pattern was observed as well as differences in survival pattern, which may reflect intrinsic differences in biological potentialities, not expressed in the "natural" history. It may be speculated, however, that such differences gain in relevance with the advancement of modern multimodality treatment and may give rise to a different therapeutic response (Stein *et al.*, 1979; Cabanillas *et al.*, 1979; Mann *et al.*, 1979; Hubbard *et al.*, 1982; v.d. Berg *et al.*, 1983). This is especially important since it has been shown that an initial complete response gives a far better prognosis than a partial or minimal response (Cabanillas *et al.*, 1979; Hermann *et al.*, 1982).

The authors wish to thank Prof. K. Lennert for the use of his extensive material and his helpful comments throughout the study. They are also thankful to Mrs. L. Kiencke for her intensive collaboration with the filing of the patients' data, to Mr. H. Wierenga for preparing the photographs, to Mr. J. Scheffers for his drawings and to Mrs. A.O. Boer for typing the manuscript.

### References

ALAVAIKKO, M. & AINE, R. (1982). The Lukes and Collins classification of non-Hodgkin's lymphomas. I. A histological reappraisal of 301 cases. *Acta Pathol. Microbiol. Immunol.* (Scand. Sect. A), **90**, 241.

CABANILLAS, F., SMITH, T., BODEY, G.P., GUTTERMAN, J.U. & FREIREICH, E.J. (1979). Nodular malignant lymphomas – factors affecting complete response rate and survival. *Cancer*, **44**, 1983.

CULLEN, M.H., LISTER, T.A., BREARLEY, R.L., SHAND, W.S., & STANSFELD, A.G. (1979). Histological transformation of non-Hodgkin's lymphoma. A prospective study. *Cancer*, **44**, 645.

DAMBER, L., LENNER, P. & LUNDGREN, E. (1982). The impact of growth pattern on survival in non-Hodgkin's lymphomas classified according to Lukes and Collins. *Pathol. Res. Pract.*, **174**, 42.

ELIAS, L. (1979). Differences in age and sex distributions among patients with non-Hodgkin's lymphoma. *Cancer*, **43**, 2540.

HERRMANN, R., BARCOS, M., STUTZMAN, L. & 4 others. (1982). The influence of histologic type on the incidence and duration of response in non-Hodgkin's lymphoma. *Cancer*, **49**, 314.

HUBBARD, S.M., CHABNER, B.A., DE VITA, D.T. & 7 others. (1982). Histologic progression in non-Hodgkin's lymphoma. *Blood*, **59**, 258.

JONES, S.E., FUKS, Z., BULL, M. & 5 others. (1973). Non-Hodgkin's lymphomas. IV. Clinico-pathologic correlation in 405 cases. *Cancer*, **31**, 806.

KIM, H. & DORFMAN, R.F. (1974). Morphological studies of 84 untreated patients subjected to laparotomy for the staging of non-Hodgkin's lymphomas. *Cancer*, **33**, 657.

LENNERT, K. (1978). Malignant lymphomas other than Hodgkin's disease. In: *Histology, Cytology, Ultrastructure, Immunology*. Springer Verlag, Berlin-Heidelberg-New York. p. 00

LENNERT, K. (1981). *Histopathology of non-Hodgkin's Lymphomas*. Springer Verlag, Berlin-Heidelberg-New York. p. 00.

LUKES, R.M. & COLLINS, R.D. (1974). Immunologic characterization of human malignant lymphomas. *Cancer*, **34**, 1488.

MANN, R.B., JAFFE, E.S. & BERARD, C.W. (1979). Malignant lymphomas – a conceptual understanding of morphologic diversity. *Am. J. Pathol.*, **94**, 105.

MOLENAAR, W.M., VAN DER BERG, H.M., HALIE, M.R. & POPPEMA, S. (1983). The heterogeneity of follicular center cell lymphomas. I. Cytohistological, immunological and enzymehistochemical aspects. *Cancer*, **52**, 2269.

MUSSHOFF, K., V. STOTZINGEN, W., SCHMIDT-VOLLMER, H. & UMBACH, H. (1981). Investigation results of a comparison made between the Kiel and Rappaport classifications of the non-Hodgkin's lymphomas, together with clinical data. *J. Cancer Res. Clin. Oncol.*, **100**, 167.

NATHWANI, B.N. (1979). A critical analysis of the classification of non-Hodgkin's lymphomas. *Cancer*, **44**, 347.

NATIONAL CANCER INSTITUTE. (1982). Sponsored study of classifications of non-Hodgkin's lymphomas. Summary of description of a working formulation for clinical usage. *Cancer*, **49**, 2112.

PATCHEFSKY, A.S., BRODOVSKY, H.S., MENDUKE, H. & 4 others. (1974). Non-Hodgkin's lymphomas: a clinicopathologic study of 293 cases. *Cancer*, **34**, 1173.

QAZI, R., AISENBERG, A.C. & LONG, J.D. (1976). The natural history of nodular lymphoma. *Cancer*, **37**, 1923.

RAPPAPORT, H. (1966). *Atlas of Tumor Pathology. Tumors of the Hematopoietic System*. Washington, DC, Armed Forces Inst. Pathol. p. 00.

RISDALL, R., HAPPE, R.T. & WARNKE, R. (1979). Non-Hodgkin's lymphoma: a study of the evolution of the disease based upon 92 autopsied cases. *Cancer* **44**, 529.

SPSS UPDATE 7–9. (1981). *New Procedures and Facilities for Releases 7–9*. (Eds. Hull & Nie) McGraw-Hill Book Company, Chicago.

STEIN, R.S., COUSAR, J., FLEXNER, J.M. & 4 others. (1979). Malignant lymphomas of follicular center cell origin in man. III. Prognostic features. *Cancer*, **44**, 2236.

VAN DEN BERG, H.M., MOLENAAR, W.M., POPPEMA, S. & HALIE, M.R. (1983). The heterogeneity of nodular follicle center cell tumors. II. Clinical follow-up of 30 patients. *Cancer* **52**, 2264.

WARNKE, R.A., KIM, H., FUKS, Z. & DORMAN, R.F. (1977). The co-existence of nodular and diffuse patterns in nodular non-Hodgkin's lymphomas. *Cancer*, **40**, 1229.